# Development of a Definition for Medical Affairs Using the Jandhyala Method for Observing Consensus Opinion Among Medical Affairs Pharmaceutical Physicians

**DOI:** 10.3389/fphar.2022.842431

**Published:** 2022-02-22

**Authors:** Ravi Jandhyala

**Affiliations:** ^1^ Medialis Ltd., Banbury, United Kingdom; ^2^ Centre for Pharmaceutical Medicine Research, King’s College University, London, United Kingdom

**Keywords:** medical affairs, definition, pharmaceutical physicians, real-world evidence (RWE), expert opinion

## Abstract

**Background**: There is currently no standard definition of medical affairs, despite its increasing importance to the pharmaceutical industry. The evolution of medical affairs necessitated the development of a standardised definition to guide policy and practice to ensure that patients’ interests remain central amid shifts that have, in the past, created fertile ground for ethical violations.

**Objectives**: The aim of this study was to use an empirical method to observe a consensus of expert opinion on the definition of medical affairs to guide policy and practice within this function.

**Methods**: In total, 11 medical affairs pharmaceutical physicians (MAPPs) completed a qualitative online survey to identify a list of key items to define medical affairs using the Jandhyala method for generating a consensus of expert opinion. Responses were coded and scored, and aggregated responses were presented to participants in a consensus round. Participants rated their agreement with each item on a 5-point Likert scale from strongly agree to strongly disagree. Indicators that reached a consensus index of >50% (CI > = 0.51) were retained. Items were categorised per previously defined medical affairs functions to determine the scope of the definition. A comparative content analysis using a previous definition identified in the literature was conducted to determine the utility of the definition generated here.

**Results**: In total, 11 MAPPs generated 15 unique items to define medical affairs. Item awareness indices ranged from 0.24 (‘communication/education’) to 1.00 (‘design/strategy’). All items had a CI of more than 0.5 and were included in the final definition. All items could be categorised per previously defined medical affairs functions. Comparative content analysis showed that our definition varied in four ways: the designation of medical affairs as a medical specialty (and its primary aim, therefore, is to protect patients), the leadership of medical affairs in medicine adoption, the generation of real-world evidence and the specification of distinct stakeholders who benefit from medical affairs.

**Conclusion**: A standard definition of medical affairs that incorporates the key principles of medical affairs as a medical specialty that leads medicine adoption and generates real-world evidence for specific stakeholders may protect and further the interests of patients by governing practice and policy.

## Introduction

While research has charted the evolution of medical affairs (Jain, 2017; Bedenkov et al., 2020; Ashkenazy, 2020) and medical affairs pharmaceutical physicians (MAPPs) (Setia et al., 2018; Sweiti et al., 2019), there is a lack of peer-reviewed research on the exact functions and scope of both the profession and the field of study, including a lack of standardised definitions. Standardised definitions are needed in the medical field to optimise research and clinical practice such that the impact of the profession on patient outcomes can be assessed and enhanced (Armstrong and Mouton, 2018). In other words, to determine and evaluate best practice guidelines in medical affairs, we must first determine and agree upon the scope of practice. Definitions determine what is measured in research and may impact the validity of results (Patino and Ferreira, 2018) as well as the ability of researchers to interpret and replicate findings (Armstrong and Mouton, 2018). As such, definitions guide both clinical practice and the research that informs it and are, therefore, needed to progress the utility of medical affairs within the pharmaceutical industry for the ultimate benefit of patients.

Previous work has addressed the functions and unique value of MAPPs to pharmaceutical companies (Jandhyala, 2022), but as yet, there has been no empirically generated definition of medical affairs. While definitions have been proposed within empirical studies on related topics (Jain, 2017; Maeda, 2021), and growing attention has been given to what changes have occurred in the role of medical affairs post-COVID-19 (Rajadhyaksha, 2020; Ghosh et al., 2021), there is no consensus on what constitutes medical affairs and its boundaries of scope. Different understandings of medical specialties may affect how they are practiced and regulated as well as healthcare policy and how training is governed (Jamoulle et al., 2017). Additionally, organisational studies of professional functions have suggested that role scope is determined by the position of the professional and the hierarchical level of the function within an organisation (Rieg, 2018). Preliminary evidence from a pilot study has suggested that medical affairs may not be prioritised within pharmaceutical companies in terms of hierarchy (Jandhyala, 2020a), despite the key role of MAPPs in satisfying distinct multiple external stakeholder requirements (Keene et al., 2020; Jandhyala, 2021a) for the successful adoption of medicines (Jandhyala, 2021b; Jandhyala, 2022).

The aim of this study was to generate a definition of medical affairs using a validated empirical method to promote the appropriate positioning of the MAPP specialty within the hierarchy in pharmaceutical companies to ensure that MAPPs are given sufficient role scope to fulfil their duty to patients, companies and their professional regulators.

## Materials and Methods

### Participants and Recruitment

A total of 13 MAPPs were recruited using convenience sampling via professional networks and invited to participate in the MAPP evidence generating programme. The programme comprised several research projects focused on the professional role of MAPPs within the pharmaceutical industry and was carried out over a 12-month period from December 2020 to December 2021. This project was carried out between October and November 2021. Over the course of the programme, two MAPPs dropped out, leaving 11 in the analysis for this project. To be included, participants had to have at least 2 years of global or regional medical affairs experience at a global pharmaceutical company with offices in the United Kingdom. There was no geographic limitation for inclusion. While 10 participants were located in the United Kingdom at the time of study, the context was global, as participants were responsible for global medical affairs as well as for a specific range of EU and US territories (Switzerland, Germany, Ireland, the Nordics, the United States and the United Kingdom). Written informed consent was obtained from all participants after providing information about the study and before the study commenced. Responses were anonymised, and consensus round list items were not identifiable to particular participants. In accordance with international regulations, ethical approval for this study was granted by King’s College London Research Ethics Committee (Reference number: MRA-21/22-26399).

### Development of a Definition for Medical Affairs Using the Jandhyala Method for Generating a Consensus of Expert Opinion

MAPPs were invited to complete an anonymous qualitative online survey about what they believed should be included in a definition of medical affairs using a consensus method known as the Jandhyala method (Jandhyala, 2020a). The Jandhyala method is a validated novel approach that is distinct from other consensus methods, such as Delphi and modified-Delphi approaches, as it contains metrics at the awareness and consensus stages to provide a quantification of participants’ awareness of, and agreement with, each list item generated (Jandhyala, 2020b). It has been used in previous work to develop and validate quality of life scales, disease-severity scales and core datasets (Damy et al., 2021; Jandhyala, 2021c) and is conducted in two rounds of online surveys, Awareness Round (1) and Consensus Round (2).

For Awareness Round 1, the online survey invited participants to provide at least three and up to 50 free-text responses, each referring to one item they believed should be included in the definition in response to the following question: What is your definition of medical affairs, including all components you feel constitute the discipline? All responses were coded by two research analysts, with discrepancies settled by the author. Participants’ survey responses received a score of one for each code they referred to. This comprised the awareness score, which showed how much knowledge each participant contributed. We constructed a definition from participants’ aggregated coded responses using standard grammatical rules as follows and presented this to participants in an anonymised online survey for Consensus Round 2:

Medical Affairs is the medical speciality that protects patients’ interests by regulating pharmaceutical company activities and leads medicine adoption through the design, implementation and communication of real-world evidence targeted to the needs of regulators, payors, prescribers, and patients.

We listed the items in the definition (participants’ aggregated codes) and asked participants to rate their agreement with the inclusion of each item in the definition on a 5-point Likert scale from strongly agree to strongly disagree. Items that attained a consensus index of >50% (CI > = 0.51) were retained in the final definition for medical affairs.

### Determining the Utility of the Definition

We generated descriptive meanings for each item according to previous findings and categorised items according to previously described MAPP functions(Jandhyala, 2022). To demonstrate the utility of the definition, we conducted a comparative content analysis (Vaismoradi et al., 2013) with the most recent existing definition we could find in the literature, which was as follows: “Medical affairs departments aim to fulfil unmet medical needs through the generation of scientific evidence and to deliver scientific value to key stakeholders and patients. Medical affairs departments aim to fulfil unmet medical needs through the generation of scientific evidence and to deliver scientific value to key stakeholders and patients. People working in medical affairs need to engage in scientific exchange activities with key opinion leaders independent of sales departments. Through these activities, medical affairs ensures that patients receive optimal medical care” (Maeda, 2021). To conduct the content analysis, we coded the definitions per the following analytic parameters: “who/what,” primary aim, primary mechanism, secondary aim, secondary mechanism, beneficiaries and outcome of activities. We assessed semantic similarities and differences across the parameters in the context of previous literature.

## Results

**FIGURE 1:**
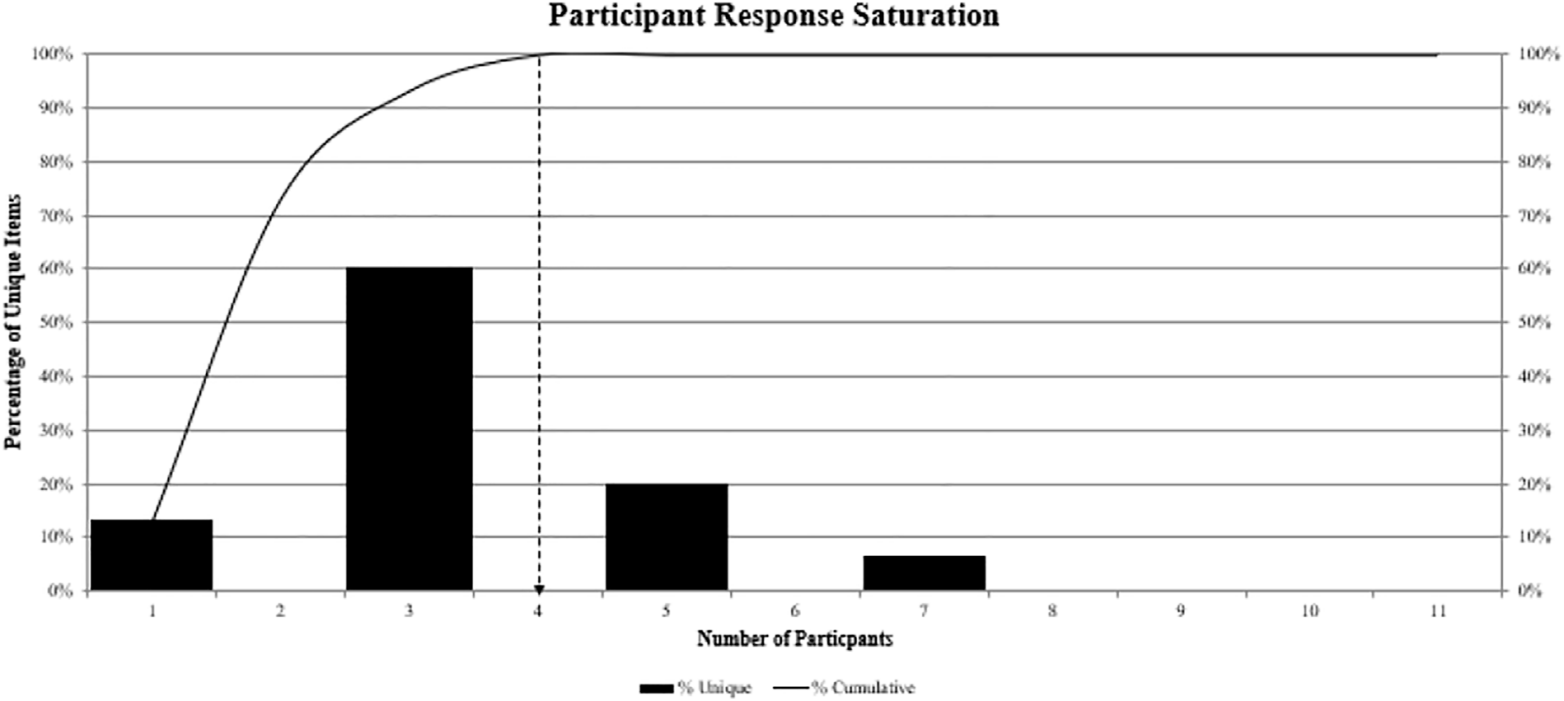
Data saturation. Bars represent the percentage of unique items generated by participants in order of entry into the study; the line represents the cumulative percentage of unique items, which was achieved by four participants in total.

In total, 15 unique items were generated during the Awareness Round 1) of the Jandhyala method (Table 1). Item awareness indices ranged from 0.24 (Item 10, “communication/education”) to 1.00 (Item 8, “design/strategy”), which all participants mentioned in Awareness Round 1. There were four items with awareness indices of less than 0.5, including Item 10 (0.24, “communication/education”); Item 7 (0.47, “adoption”); Item 11 (0.41, “real-world evidence”) and Item 12 (0.47, “regulator”). In Consensus Round 2, five items received unanimous agreement for inclusion in the definition, which were Item 1, “medical”; Item 2, “protecting patient interests”; Item 3, “regulating pharma company activities”; Item 7, “adoption” and Item 11, “Real-world Evidence” (see Table 1 for item details). All other items received consensus scores of 0.91, which meant that all items met the threshold for inclusion in the final definition. In the present study, Participants 1, 3, 5, and 7 provided unique items in Awareness Round 1 (Figure 1). Therefore, data saturation, defined as the point at which no further unique items were generated, was reached by a total of four participants after seven participants had provided responses. The final definition for medical affairs was as follows:

**TABLE 1:** Awareness and consensus scores for items in the medical affairs definition.

Item	Statements	A	C
Item 1	Medical [used to denote a key characteristic that is not commercial, sales or market access and therefore non-promotional]	2	1
Item 2	Protecting patient interests [espousing *primum non nocere* and occupies the primary position in the definition in recognition of its fundamental importance to the medical profession]	2	1
Item 3	Regulating pharma company activities [through exercising the power of veto conferred on the individual carrying out a Medical Affairs function including medical examination and certification duties]	2	1
Item 4	Speciality [therefore, MAPPs are subject matter experts]	2	2
Item 5	Leading [emphasises the ‘leadership’ conferred on someone required to make decisions that can only be fulfilled by an individual with medical experience]	2	2
Item 6	Medicine [refers to a specific prescription-only medicine]	2	2
Item 7	Adoption [refers to the progression of the medicine through the gatekeeper stakeholders referred to later before finally reaching a patient who then receives a benefit]	3	1
Item 8	Design/Strategy [generating a plan for evidence-based on multiple stakeholder needs to ensure optimal adoption of the medicine]	1	2
Item 9	Implementation/Generation [involvement in generating the evidence in the plan]	2	2
Item 10	Communication/Education [communicating results of completed studies (clinical trials and RWE) via various channels]	4	2
Item 11	Real-world Evidence [see Real-world Evidence to be used in conjunction with this medical affairs definition]	3	1
Item 12	Regulator [a gatekeeper stakeholder that the medical affairs function is required to provide evidence to in order to facilitate the adoption of a medicine]	3	2
Item 13	Payor [a gatekeeper stakeholder that the medical affairs function is required to provide evidence to in order to facilitate the adoption of a medicine]	2	2
Item 14	Prescriber [a gatekeeper stakeholder that the medical affairs function is required to provide evidence to in order to facilitate the adoption of a medicine]	2	2
Item 15	Patient [the ultimate beneficiary of a successfully adopted medicine and the subject of the real-world evidence used to facilitate the adoption of a prescription-only medicine]	2	2

A: awareness score; C: consensus score.

“*Medical Affairs is the medical speciality that protects patients’ interests by regulating pharmaceutical company activities and leads medicine adoption through the design, implementation and communication of real-world evidence targeted to the needs of regulators, payors, prescribers and patients*”.

Items of the medical affairs definition could be categorised into four previously defined MAPP functions (Table 2) (Jandhyala, 2022). Content analysis showed that the consensus-generated definition varied from previous definitions in four key ways: the designation of medical affairs as a medical specialty, the leadership of medical affairs in medicine adoption, the generation of real-world evidence and the specification of distinct stakeholders who benefit from medical affairs (Table 3). The previous definition viewed medical affairs as a department in which non-medically trained staff work and failed to specify its regulatory function, referring instead to its independence from commercial functions. The definitions were similar in that they both referred to the generation of scientific evidence for the benefit of patients and stakeholders, but the consensus-generated definition was more specific in that it provided information on the type of evidence medical affairs is concerned with and the distinct stakeholders to whom this evidence is conveyed.

**TABLE 2:** Medical affairs definition items categorised per previously defined MAPP activities^8^.

Activity	Items
Protecting patients’ interests	1, 2, 3
Medicine adoption and regulation	4, 5, 6, 7
Evidence generation	12, 13, 14, 15
Gatekeeper stakeholder engagement	8, 9, 10, 11

**TABLE 3:** Comparative content analysis of medical affairs definitions.

Analytic parameter	Consensus-generated definition	Previous definition (Maeda, 2021)
Who or what	*Medical Affairs is the medical speciality*	Medical affairs departments
		People working in medical affairs
Primary aim	*that protects patients’ interests*	aim to fulfil unmet medical needs
Primary mechanism	*by regulating the activities of a pharmaceutical company*	through the generation of scientific evidence
Secondary aim	*and leads medicine adoption*	need to engage in scientific exchange activities with key opinion leaders independent of sales departments
Secondary mechanism	*through the design, implementation and communication of real-world evidence*	to deliver scientific value to
Beneficiaries	*targeted to the needs of the regulator, payor, prescriber, and patient*	key stakeholders and patients
Outcome of activities		Through these activities, medical affairs ensures that patients receive optimal medical care

## Discussion

There is a need for a standardised definition of medical affairs to optimise research, clinical practice, patient outcomes, policy and governance (Armstrong and Mouton, 2018; Patino and Ferreira, 2018). We used the Jandhyala method to define medical affairs by the consensus of 11 MAPPs, who generated 15 unique items for inclusion in the definition, which was as follows:

Medical Affairs is the medical speciality that protects patients’ interests by regulating pharmaceutical company activities and leads medicine adoption through the design, implementation and communication of real-world evidence targeted to the needs of regulators, payors, prescribers and patients.

Items could be categorised into previously defined MAPP functions (Jandhyala, 2022), and the definition varied from previous definitions in four key ways: the designation of medical affairs as a medical specialty, the leadership of medical affairs in medicine adoption, the generation of real-world evidence and the specification of distinct stakeholders who benefit from medical affairs.

Of the 15 items generated by participants for the definition of medical affairs, they were most aware of “design/strategy” and least aware of “communication/education.” This could be due to the traditional separation of medical affairs and commercial functions within pharmaceutical companies, whereby drug promotion is isolated from scientific interests for ethical reasons (Jacob, 2018). However, medical affairs may be increasingly responsible for the communication and dissemination of scientific knowledge, especially after the COVID-19 pandemic (Ghosh et al., 2021). This could explain why MAPPs agreed to include “communication/education” in the final definition despite being less aware of it initially. Participants unanimously agreed on the inclusion of five items: “medical,” “protecting patient interests,” “regulating pharma company activities,” “adoption,” and “Real-world Evidence.” All other items were agreed upon by 10 of the 11 participants. Therefore, the definition was robustly supported by consensus. All items could be categorised into four previously defined MAPP functions: protecting patients’ interests, medicine adoption and regulation, evidence generation and gatekeeper stakeholder engagement (Jandhyala, 2022), which provided confidence in the reliability and validity of our findings.

Our definition varied from a previous definition (Maeda, 2021) in four ways: the designation of medical affairs as a medical specialty, the leadership of medical affairs in medicine adoption, detail regarding the generation of real-world evidence and the specification of distinct stakeholders who benefit from medical affairs. The lack of designation of medical affairs as a medical specialty is common in published literature, with some perspectives viewing it as a partnership with physicians rather than a speciality comprised of physicians (Bedenkov et al., 2020) and others as a partnership between non-physician medical staff and medical communications (Jain, 2017). Most viewed the specialty as being staffed primarily by non-physicians who are not regulated by the General Medical Council (GMC) or other physician governance bodies (Jain, 2017; Bedenkov et al., 2020; Maeda, 2021). Historically, the pressures of commerciality within pharmaceutical companies resulted in ethical violations when medical affairs functions, such as the communication of scientific knowledge, were poorly regulated (Maeda, 2021). These issues were resolved by separating communication and scientific functions (Jandhyala, 2020a; Maeda, 2021); however, the increasing need for medical affairs to communicate and disseminate scientific knowledge responsibly (Ghosh et al., 2021) may necessitate this kind of professional regulation, including accountability to the GMC with the possibility of sanctions, now more than ever.

Further, the designation of medical affairs as a medical specialty affords specific protections not otherwise provided by the function. For example, medical specialities adhere to the professional standard of *primum non nocere*, which protects patients as a matter of principle. Despite calls for more patient-centric medical affairs, this has not been addressed systematically or achieved to date, and existing frameworks suggest that patient-centricity should be a guiding principle but offer no way of ensuring this (Bedenkov et al., 2020; Ashkenazy, 2020). Individual moral identity has been identified as a protective factor against unethical pro-organisational employee behaviour (Kim et al., 2018) such as the ethical violations that occur when profit is placed above patients’ interests. However, variation in moral identity between individuals (Körner et al., 2020) suggests that the protection of patients would be enacted to varying standards across medical affairs departments without adherence to a moral code operationalised via professional ethical governance such as that afforded by defining medical affairs as a medical specialty.

Business leadership skills have been recognised as increasingly important in medical affairs (Bedenkov et al., 2020), but there is a dearth of research on the processes this function leads. This was reflected in the low awareness score for “adoption” in this study, which referred to the process of medicine adoption through pharmaceutical gatekeeper stakeholder subsystems (Jandhyala, 2021a), which received unanimous agreement in the consensus round. This suggested a need for further evidence generation on medicine adoption and the education and training of MAPPs and others who work in medical affairs on this process. Our definition also highlighted the fact that the medical affairs generates real-world evidence to benefit distinct stakeholders. Previous definitions refer to scientific evidence and stakeholders without distinction (Bedenkov et al., 2020; Maeda, 2021). However, real-world evidence is generated through specific methods and in specific populations and contexts that differentiates it from other types of evidence (Körner et al., 2020). Additionally, stakeholders have distinct evidentiary needs (Keene et al., 2020; Jandhyala, 2021b) that must be fulfilled for successful medicine adoption (Jandhyala, 2021c). Specifying the type of evidence and each stakeholder involved in medicine adoption within the definition of medical affairs may focus research (Patino and Ferreira, 2018) and practice and benefit policy and governance (Jamoulle et al., 2017; Armstrong and Mouton, 2018) in this function to enhance outcomes for both patients and pharmaceutical companies.

## Limitations

First, our definition was limited by the small sample size and geographical locations. However, it was unlikely for the small sample size to have affected our findings, as we reached saturation after the participation of seven MAPPs. Additionally, while our definition was applicable in a global context due to MAPP operation at regional and global levels, MAPPs at different pharmaceutical companies may vary in the way they define medical affairs, and this could be determined by further research. It may be helpful to determine whether awareness of the medical affairs function’s role in medicine adoption is low in the general population of MAPPs, as this may account for the lack of understanding of this aspect of medical affairs within research and practice as well as providing a possible avenue for change. Second, our definition was limited by being written only from the point of view of MAPPs. This may have been quite important, as the variety of other roles that work in medical affairs, such as medical science liaisons, may have a different perspective informed by their own role. However, our findings suggested that predominant ideas about medical affairs were more reflective of the functions of other roles and not physicians, even though they receive specialist training in medical affairs, are professionally regulated (and thus, accountable in their duty to patients) and to some extent, fulfil a regulatory function in terms of overseeing pharmaceutical activities to ensure that commercial interests are guided by ethical and scientific principles, which protect patients. Additionally, definitions in the literature provided by non-MAPPs did not add anything to our definition but left out key aspects needed to ensure safe and effective medical affairs practice. Therefore, a definition of medical affairs from the point of view of MAPPs was both needed and justified.

## Conclusion

Our study generated the first empirical definition of medical affairs as “the medical speciality that protects patients’ interests by regulating pharmaceutical company activities and leads medicine adoption through the design, implementation and communication of real-world evidence targeted to the needs of regulators, payors, prescribers and patients.” Of the 15 items generated by participants, five were agreed unanimously for inclusion in the definition, and the rest were agreed by 10 of the 11 MAPPs. Our definition varied from existing definitions in four key ways: the designation of medical affairs as a medical specialty (and its primary aim, therefore, is to protect patients), the leadership of medical affairs in medicine adoption, the generation of real-world evidence and the specification of distinct stakeholders who benefit from medical affairs. These areas define the role scope of MAPPs and govern the key principles recognised as necessary in medical affairs practice. The use of this definition may protect and further the interests of patients by enhancing research and practice in medical affairs.

## Data Availability

The original contributions presented in the study are included in the article/Supplementary Material, further inquiries can be directed to the corresponding author.
